# Cause of Death in Patients in Radiation Oncology

**DOI:** 10.3389/fonc.2021.763629

**Published:** 2021-10-21

**Authors:** Justus Domschikowski, Karoline Koch, Claudia Schmalz

**Affiliations:** ^1^Department of Radiation Oncology, University-Hospital Schleswig-Holstein, Kiel, Germany; ^2^Department of Pathology, University Hospital Schleswig-Holstein, Kiel, Germany

**Keywords:** cause of death, medical autopsy, radiation oncology, discrepancies, oncology, palliative sedation

## Abstract

**Background:**

The accurate attribution of death in oncologic patients is a difficult task. The patient’s death is often attributed to his or her underlying cancer and therefore judged as cancer-related. We hypothesized that even though our patient’s cancers were either advanced or metastatic, not all patients had died simply because of their cancer.

**Methods:**

A total of 105 patients were included in this retrospective analysis. Patient data were collected from digital and paper-based records. Cause of death was assessed from death certificate and compared to the medical autopsy reports. Discrepancies between premortem and postmortem diagnoses were classified as class I and II discrepancies.

**Results:**

Of 105 patients included, autopsy consent was obtained in 56 cases (53%). Among them, 32 of 56 were palliatively sedated, and 42/56 patients died cancer-related as confirmed by autopsy. The most common cause of death by autopsy report was multiorgan failure followed by a combination of tumor and infection, predominantly lung cancer with pneumonia. Here, 21/56 cases (37%) showed major missed diagnoses: seven cases showed class I, 10 class II, and both discrepancies. The most commonly missed diagnoses in both categories were infections, again mainly pneumonia.

**Conclusions:**

Cancer was the leading cause of death in our study population. A quarter of the patients, however, did not die due to their advanced or metastatic cancers but of potentially curable causes. We therefore conclude that it is important to consider competing causes of death when treating palliative cancer patients. In a palliative setting, the treatment of a potentially curable complication should be discussed with the patients and their families in a shared decision-making process. From our experience, many patients will decline treatment or even further diagnostics when given the option of best supportive care.

## Introduction

As radiation oncologists, we accompany patients in the last stages of their illness. Occasionally, we have to ask ourselves: what did our patient die of and was their death related to our therapy? One way to find a clearer, but far from absolute, answer to this fundamental question is through medical autopsy.

Historically, the medical autopsy has been one of the foundations for modern understanding of diseases and their causes. Shojania et al. (1, 2) and Goldman et al. (3), in a comprehensive review of autopsy series, proposed a classification system for discrepancies between premortem and postmortem diagnoses. It is based on whether a treatment, had the diagnosis been known before death, would have had an impact on patient survival. They also showed a correlation between a high autopsy rate and a lower number of discrepancies. Therefore, autopsy was declared a quality assurance tool in medicine. Autopsy rates have steadily declined in the past (4–8), and numerous reasons have been suggested for this (9–11), ranging from a change in the pathologist’s workload—from macroscopic to microscopic—to a change in the physician’s perspective of the possible gain from an autopsy. Various options have been tried to increase autopsy rates, such as a better compensation for the pathologist or the incorporation in quality criteria for oncologic centers, but so far, none has proven to be clearly effective, as autopsy rates in Germany have not increased in recent years (10).

Another critical area is the determination of the immediate cause of death by the clinician. The clinician who determines the patient’s death is usually required to complete a form indicating the patient’s underlying disease and immediate cause of death. The correct assignment of cause of death can vary widely depending on the physician’s training and experience (10, 12–16). This may result in overreporting or underreporting of cancer-related mortality (16–18). Therefore, systematic trainings concerning the special requirements for filling out death certificates seem to be important.

As autopsy rates are low and the correct attribution of the cause of death may vary, the aim of this retrospective case series is to describe the cause of death of patients in radiation oncology as confirmed by medical autopsy. Although most patients had either locally advanced or metastatic disease, we hypothesized that not all had died due to their underlying cancer.

Because we considered our autopsy rate to be unusually high compared with the estimated overall autopsy rate, we examined our data for a possible association between autopsy consent and patient characteristics.

## Methods

Institutional ethics committee approval was obtained for this retrospective study. The sole inclusion criterion was death at the ward of the Department of Radiation Oncology at the University Hospital Schleswig-Holstein in Kiel between 2013 and 2017. Both paper-based and digital patient records were assessed, and data were systematically captured.

Diagnostic discrepancies were classified as class I or class II according to Shojania et al. (1), Goldman et al. (3), and the Bundesaerztekammer (9). A finding was judged as class I discrepancy if it was related to the cause of death and, if treated, could have had a positive effect on the patients’ survival. An unknown diagnosis with unlikely influence on the patient’s survival even if it would have been treated correctly was judged as class II. The classification was done independently by two clinician reviewers. In case of disagreement, cases were discussed with a pathologist to find a consensus.

Medical autopsy reports were reviewed by a pathologist. The immediate cause of death was categorized as cancer-related as a combination of cancer with other underlying diseases or as unrelated to cancer.

Descriptive statistical analysis was performed using Microsoft Excel. Correlation between variables for linear correlation was calculated using Pearson’s correlation coefficient, and its significance was interpreted *via* critical value table. Binomial tests and chi-square tests were used to further calculate the significance between variables.

## Results

Between May 2013 and April 2017, 2,779 cancer patients were treated at our ward. Four percent, 105 patients, died in the radiation oncology ward and were included in this retrospective study. **Table 1** gives an overview of the baseline characteristics of, firstly, the overall study population, second, of those referred to autopsy, and, third, of those who did not consent to autopsy.

**Table 1:** Study population overview and patient characteristics.

Patient Characteristics			
	Overall	Autopsy	No Autopsy
	(n = 105)	(n = 56)	(n = 49)
Female	42 (40%)	20 (36%)	22 (45%)
Male	63 (60%)	36 (64%)	27 (55%)
Age, years, median	70.3	70.06	71.3
(IQR)	(64–76)	(64–75)	(66–78)
Survival after first diagnosis, months, median	3.9	4.9	3.9
(IQR)	(1.9–15.9)	(1.9–15)	(1.9–15.9)
Average length of stay, days (IQR)	14	11.5	15
	(8–22)	(7–21)	(11–23)
**Treatment Intent**			
Curative, Adjuvant, Definitive	12 (11%)	4 (7%)	8 (16%)
Palliative	87 (83%)	48 (85%)	39 (80%)
No Radiotherapy Intended	6 (5%)	4 (7%)	2 (4%)
**Top 3 Tumor Entities**			
Lung	48 (46%)	29 (51%)	19 (39%)
Breast	10 (10%)	5 (9%)	5 (10%)
Bladder	5 (5%)	2 (4%)	3 (6%)
Palliative Sedation	51 (48.6%)	32 (57%)	19 (39%)

n, number; IQR, interquartile range. Shown are the characteristics of our overall study population and of those who did or did not undergo medical autopsy.

Treatment intention was palliative for 90% of patients; only 12 patients were treated with curative intent. Three Gray per treatment fraction was prescribed in half of the patients, and cerebral and spinal metastases were most frequently irradiated. The prescribed radiation treatment was completed in 57% of the treated patients. Forty-three percent had to be discontinued prematurely or were not started at all. When irradiation had to be terminated, patients were treated with best supportive care. The average length of hospital stay was 14 days, 5 days longer than the German average (19).

The majority of cancers were either locally advanced (T3/4, 44%) or metastatic (M1, 47%). Lung cancer was most common followed by breast and bladder cancer. Survival after first diagnosis was very limited, with two-thirds of patients having died within the first 6 months after initial diagnosis. Before their death, half of the study population was treated with palliative sedation.

**Figure 1:**
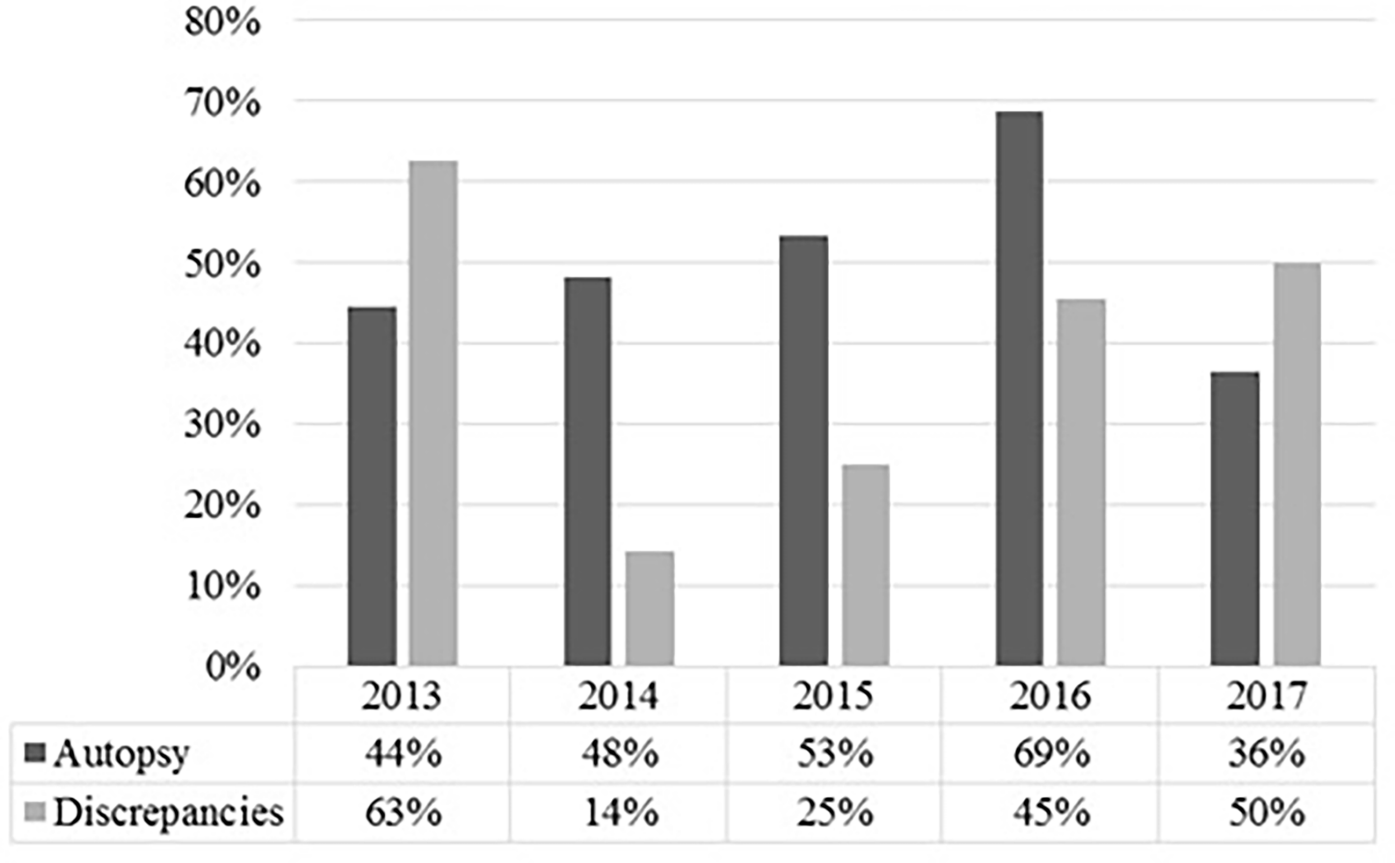
Shown are the autopsy rate and the rate of discrepancies per medical autopsy in percent for the respective years. It shows that a high autopsy rate does not necessarily correlate with a lower rate of discrepancies in oncologic patients.

In every case, relatives of our patients were asked for their consent to an autopsy of their next of kin; in 56 cases, consent was obtained, representing an autopsy rate of 53.3%. The variation in autopsy rate in correlation with diagnostic discrepancies over the observed time span is shown in **Figure 1**.

Patient characteristics such as gender, age, length of hospital stay, palliative therapy, or time of onset of illness were examined for their influence on the consent rate and tested statistically. Of the characteristics studied, palliative sedation of more than 1 day showed a statistically significant impact (p <.05) on consent to autopsy.

After autopsy, a direct association with the underlying cancer was found in most cases (26 out of 56), a combination of cancer with another advanced disease was thought to be the cause in 16 cases, and no association with the treated cancer was found in 14 cases. In the tumor and infection subcategory as a combined cause of death, pneumonia was responsible for infection in eight of 10 cases. Five of these eight cases were lung cancer. And 80% of the cases unrelated to cancer had a metastatic tumor.

The relationship between disease and cause of death can be seen in **Table 2**.

**Table 2:** Shown are the immediate causes of death as confirmed by pathologists.

Immediate Cause of Death per medical autopsy review		
**Cancer-related**		
**(n = 26)**		
	Multiple organ failure	15
	Central nervous regulatory failure	4
	Liver failure	2
	Local complications (e.g., bleeding)	5
**Combined causes**		
**(n = 16)**		
	Tumor and infection	10
	Tumor and cardiac complications	4
	Tumor and embolism and pneumonia	1
	Upper GI and cardiovascular	1
**Unrelated to cancer**		
**(n = 14)**		
Cardiovascular system		
	Cardiac infarction	2
Pulmonary complications		
	Pulmonary embolus	2
	Respiratory failure	1
Infections	Pneumonia	1
	Meningitis	1
	Sepsis	3
	Myocarditis	1
Major bleeding complications		
	Cerebral	1
	Upper gastrointestinal	2

Findings were categorized by a pathologist as cancer-related, as a combination of cancer and other disease or as unrelated to the underlying cancer.

Additionally, six autopsies (10%) revealed unknown secondary tumors: there were two cases of gastrointestinal stroma tumors, one additional pulmonary cancer, one rectal cancer, one meningioma, and one follicular thyroid cancer. Nine cases (16%) presented unknown progressive disease. Three (5%) showed cardiac metastases of different cancers (one non-small cell lung cancer, one Merkel cell carcinoma, and one malignant solitary fibrous tumor).

Clinically, multiorgan failure was both the most commonly suspected cause of death (37 out of 105) and pathologically most commonly found (15 out of 56).

Regarding our accuracy in predicting the correct immediate cause of death, there was a discrepancy between the physician’s judgment and the autopsy result in 33 cases (58.9%). Out of 56 autopsies, 21 cases (37%) showed major missed diagnoses. Seven cases showed class I, 10 class II, and four both discrepancies. Most common discrepancies in both categories were infections led by pneumonia followed by bleeding complications in class I discrepancies and cardiopulmonary complications in class II. Class I discrepancies were equally distributed between locally advanced and metastatic patients (five advanced, six metastatic). Class II discrepancies occurred more frequently in metastatic patients (eight patients) than in locally advanced patients (three patients). **Table 3** gives an overview of the findings regarding class I and class II discrepancies.

**Table 3:** Shown are the class I and II discrepancies between premortem and postmortem diagnoses.

Class I and II Discrepancies			N
**Class I**	Infections	Pneumonia	2
(n = 11)	(n = 5)	Meningoencephalitis	1
		Sigmadiverticulitis	1
		Urosepsis	1
	Cardiopulmonary complications	Pulmonary embolism	1
	(n = 1)		
	Cardiac complications	Cardiac infarction	2
	(n = 2)		
	Major bleeding	Upper gastrointestinal bleeding	2
	(n = 3)	Cerebral	1
**Class II**	Infections	Pneumonia	6
(n = 11)	(n = 7)		
		Endocarditis	1
	Cardiopulmonary complications	Pulmonary embolism	2
	(n = 3)	Portal vein thrombus	1
	Bleeding	Subdural hematoma	1
	(n = 1)		

n, number.

## Discussion

In this study, we found that nearly half of the hospitalized patients at our ward had died of cancer-related causes and just over a quarter had died of a combination between an advanced internal disease and their cancer, leaving another quarter of cancer-unrelated deaths. The leading causes of death as confirmed by pathologists were multiple organ failure due to high tumor burden and infection in combination with cancer, usually pneumonia in lung cancer patients. Since most patients had either locally advanced tumors or metastatic disease, multiorgan failure as the most common cause of death was not unexpected. As in the general population, infections played another major part in the death of our patients (20) and were the second most common cause of death combined with underlying cancer and also the most common major missed diagnosis. Two population analyses showed a similar result; one analysis of colorectal cancer patients by Feng et al. (21) and one of renal cell carcinoma patients by Wang et al. (22). They showed that although cancer was still the most common cause of death, patients with advanced cancers had a higher all-cause mortality in the long term compared to the general population. Hence, they are more vulnerable to infections and other diseases. Furthermore, Feng et al. (21) described that 51.3% of their observed population had died colorectal cancer–related, which is similar to our rate of 46% directly cancer-associated deaths and 38% had died unrelated to cancer. Wang et al. (22) describe a lower rate of 28% non-cancer-related death, closer to our pathologically confirmed rate of 25%. Thus, to prolong survival, not only the treatment of cancer but also the prevention of non-cancer-related death, e.g., from infection, should be considered, as cancer is not the only cause of death even in very advanced or metastatic disease.

When evaluating a deteriorating palliative patient, we suggest searching for early signs of infection or bleeding complication first, as they were the most commonly missed major diagnoses. Interestingly, the distribution of class I discrepancies, i.e., those that would have had an impact on patient survival, was similar between metastatic and locally advanced patients. The difficulty in finding early signs, however, is to distinguish between laboratory values caused by the advanced cancer and those caused by infection. Elevated infection parameters do not always mean an underlying infection, and low blood counts are also a common phenomenon in oncological patients. While our data did not offer a solution to this problem, it changed, however, our clinical practice when treating hospitalized palliative patients, as we started to offer antibiotics earlier to patients. For example, if a patient is admitted with high infection markers that were judged only as cancer–related, we would nonetheless start an antibiotic therapy regimen. After 3 days, we reevaluate the patient’s general condition and the laboratory parameters and decide if the antibiotic treatment will be continued or stopped. From our experience, most patients benefit from this early antibiotic treatment. This retrospective case series, however, did not evaluate the premortem vital signs and laboratory values of our patients. Hence, there are no hard data so far to back up our clinical experience.

In our data analysis, we found an unusually high consent rate for autopsy of 53%. Compared with the estimated autopsy rate, rates are not published centrally in Germany and thus can only be estimated; it is nearly 10 times higher. Other series that systematically offered autopsies, either as questionnaire studies or in real-life situations, also had a similarly high consent rate of above 50% (23–26). This offers a possible answer to the question of why autopsy rates are generally low: autopsies may not be systematically offered to patients’ next of kin. To find other explanations for the low overall autopsy rate, other questionnaire studies (27–29) examined the practice of asking for consent or the availability of teaching materials for physicians regarding autopsies. The study results were similar. Even though physicians generally valued autopsies as an important medical procedure, most hospitals did not provide teaching materials to their residents. Furthermore, clinicians generally did not receive adequate training on how to deal with relatives. The majority of clinicians did not have a clear picture of the procedure of a medical autopsy. Therefore, we concluded that although autopsies are highly valued by physicians and relatives alike, many physicians lack the training in dealing with relatives or are unaware of the possible benefits of an autopsy. As a result, autopsies are offered less frequently. This claim is supported by a prospective study by Waidhauser et al. (30). They demonstrated that by systematically educating residents and offering autopsy as a last medical measure, a significantly higher autopsy rate can be achieved.

Regarding the consent rate in relation to patient characteristics, our data showed a strong positive correlation of palliative sedation and the consent to autopsy by the patients’ relatives. Retrospective data, however, lack the ability to further explain this statistical relationship. A possible explanation may be a closer patient–caregiver–relative relationship if a patient receives a palliative sedation. Before administering palliative sedation, we conduct a shared decision-making process with the patient, their relatives, and the treating team of nurses, physicians, and psycho-oncologists. The ensuing conversations between practitioners and relatives inevitably lead to a closer relationship. More data are needed, however, to further evaluate this special patient–caregiver relationship and the possible impact of an autopsy on the patients’ relatives and the treating team. In this regard, a prospective study is planned at our hospital.

The rate of major discrepancies (37%) in our study was within reported range for oncologic patients (14, 30–33). Moreover, in the observed years, a high rate in autopsy did not necessarily correlate with a low rate in major discrepancies, as reported in a large review (1). This missing correlation was also described by Waidhauser et al. (30). One reason may be the small sample size and short period of our and their study. Another possible explanation is the palliative situation of most of our patients. Nearly half of the evaluated patients had received palliative sedation and thus had actively decided against further diagnostic procedures, creating a diagnostic gap between the last diagnostic procedure and their death. For example, if a patient develops an infection while under palliative sedation, the attending physician usually is not aware of it because further diagnostics are not performed. In addition, some patients actively refuse treatment, such as antibiotics, even if there is no clinical doubt in the diagnosis. The diagnostic gap makes it difficult for the attending physician to correctly determine the cause of death and makes it nearly impossible to avoid minor and major discrepancies. Furthermore, the classification of minor and major discrepancies does not take into account a patient‘s will. The most personal and desperate will of a person in a palliative situation to forgo diagnostics may lead to this diagnostic gap. To address this, it might be helpful to add another category of “unavoidable discrepancies.”

As we analyzed the survival data of our study population, we found that the survival time in the overall population was relatively short, just under 4 months from initial diagnosis. This short survival is most likely a result of the selection bias of the patient population, since only hospitalized patients were included. There is a variety of scores evaluating the prognosis of patients receiving palliative radiotherapy (34–36). For example, Zaorsky et al. (35) created a scoring system based on a National Cancer Database query of over 64,000 patients. They developed their algorithm on half of the patients and validated it with the other half so that it showed a high reliability in predicting survival at 1 year. Their model was based on the location of metastasis, primary tumor, age, sex, and comorbidity scores. Patients in our study population were unable to receive outpatient radiotherapy and had to be hospitalized for treatment. This may be considered as a surrogate for a low Eastern Cooperative Oncology Group (ECOG) Score or a high Charlson Comorbidity Score. Furthermore, their underlying diseases were metastatic or very advanced. These parameters alone, when entered into the aforementioned scoring system, are associated with a very low life expectancy for patients receiving palliative radiotherapy for metastases. In our opinion, this is a possible explanation for the short survival in most of our patients.

## Limitations

As with many studies, this retrospective analysis has its weaknesses. This is only a single-institute retrospective analysis of a small subgroup of oncologic patients who were hospitalized for radiotherapy. Thus, it is questionable whether cause of death data can be extrapolated on the general population of patients in radiation oncology.

Secondly, even for pathologists, the cause of death in patients with advanced cancer can be difficult to determine with an inter-person variability. However, all retrospective autopsy studies are confronted with this problem. A solution in a prospective setting would be to determine which pathologist carries out the autopsy to receive a more homogeneous result.

Third, the classification for major discrepancies does not consider the patients’ will. Thus, there is a chance of overreporting discrepancies if the classification system is strictly followed. We suggest that if a patient freely refuses further therapy or diagnostics, the autopsy results should be scored as new diagnostic findings rather than discrepancies. The scoring system for missed diagnoses should receive an additional category to increase the precision of describing discrepancies, especially in palliative patients.

## Data Availability Statement

The raw data supporting the conclusions of this article will be made available by the authors without undue reservation.

## Ethics Statement

The studies involving human participants were reviewed and approved by Ethik Komission der Medizinischen Fakultaet der CAU zu Kiel. Written informed consent for participation was not required for this study in accordance with the national legislation and the institutional requirements.

## Author Contributions

Conceptualization, JD and CS. Methodology, JD. Investigation, JD, CS, and KK. Data curation, JD. Writing—original draft preparation, JD. Writing—review and editing, JD, CS, and KK. Visualization, JD. Supervision, CS. Project administration, CS. All authors have read and agreed to the published version of the manuscript.

## Funding

We acknowledge financial support by Land Schleswig-Holstein within the funding program “Open Access Publikationsfonds.”

## Conflict of Interest

The authors declare that the research was conducted in the absence of any commercial or financial relationships that could be construed as a potential conflict of interest.

## Publisher’s Note

All claims expressed in this article are solely those of the authors and do not necessarily represent those of their affiliated organizations, or those of the publisher, the editors and the reviewers. Any product that may be evaluated in this article, or claim that may be made by its manufacturer, is not guaranteed or endorsed by the publisher.
